# Transforming Aging: Increased Access to Care Minimizes Rural and Urban Differences in Cognitive Change

**DOI:** 10.1093/geroni/igab046.1856

**Published:** 2021-12-17

**Authors:** Timothy Ly, Rebecca Allen, Barbara Jackson, Amy Albright, John Bell, Dana Carroll, Anne Halli-Tierney

**Affiliations:** 1 University of Alabama, University of Alabama, Alabama, United States; 2 University of Alabama, Tuscaloosa, Alabama, United States; 3 The University of Alabama, Tuscaloosa, Alabama, United States; 4 VA Maine HCS, Gorham, Maine, United States; 5 Auburn University Harrison School of Pharmacy, Tuscaloosa, Alabama, United States

## Abstract

Rural-urban disparities in cognitive health outcomes, such as greater prevalence of cognitive decline among rural-dwelling older adults, have been linked to inequity in access to care. However, few studies have demonstrated whether longitudinal increased access to care may mitigate such disparities. This paper presents data from ongoing systematically collected behavioral health data on new and returning patients at an interdisciplinary geriatrics clinic at the University of Alabama Medical Center. The aim of this study was to determine baseline predictors of cognitive change across three annual visits (n = 42, mean age of 75.63 years (SD = 9.15)). Adjusting for baseline cognitive status, baseline subjective health literacy, and baseline depression and anxiety, results from a univariate ANCOVA showed that age at first visit (B = -.024, 95% CI [-.041, -.008], t(35) = -2.990, p = .005) and rural-urban status (B = .555, 95% CI [.123, .988], t(35) = 2.608, p = .013) predicted cognitive change at timepoint three (T3). Specifically, individuals from rural areas were less likely to experience cognitive decline and scored .555 points better than individuals from urban areas on cognitive screeners at T3 compared with baseline cognitive status. These results suggest that increased access to and utilization of care may ameliorate traditional disparate rates of cognitive decline between rural- and urban-dwelling older adults. Moreover, behavioral health screenings in primary geriatrics clinic care may help identify patient cognitive needs and facilitate integrated care through combined medical, pharmacological, and behavioral interventions to promote positive cognitive health outcomes.

